# Epidemiology of progressive intellectual and neurological deterioration in UK children

**DOI:** 10.1111/dmcn.70008

**Published:** 2025-09-29

**Authors:** Christopher M. Verity, Polly J. Maunder, Anne Marie Winstone, Suvankar Pal

**Affiliations:** ^1^ The PIND Research Group Addenbrooke's Hospital Cambridge UK; ^2^ The National Creutzfeldt–Jakob Disease Research and Surveillance Unit University of Edinburgh Edinburgh UK

## Abstract

**Aim:**

To study the neurodegenerative diseases that cause progressive intellectual and neurological deterioration (PIND) in children in the UK.

**Method:**

This active prospective epidemiological study asked UK paediatricians to notify all childhood cases of PIND via the British Paediatric Surveillance Unit. Clinical data were obtained using a questionnaire or via a site visit. An independent PIND study Expert Group classified the cases.

**Results:**

Between May 1997 and April 2024 (27 years), 2373 children with PIND were identified who had an underlying diagnosis to explain their deterioration. There were six cases of variant Creutzfeldt–Jakob disease plus 2367 children (1265 males, 1102 females) with other diseases. The lifetime risk of having a diagnosed disease causing PIND was 0.1 in 1000 live births. Asian British children made up 28.6% of the 2183 cases with known ethnicity. Excluding variant Creutzfeldt–Jakob disease, diagnosed children had 259 diseases, identified before death in 99% of children (only 39 were known to have had postmortems). Increasingly, diagnosis was made using genetic studies. Sixty‐one per cent (157 of 259) of the diseases were inborn errors of metabolism, affecting 78% of diagnosed children. There were 43 lysosomal diseases.

**Interpretation:**

This unique epidemiological study of many rare childhood neurodegenerative diseases provides valuable practical information about the presentation, clinical features, and inheritance of these complex disorders.

## Graphical abstract

This study of PIND in UK children was carried out via the British Paediatric Surveillance Unit from 1997 to 2024. It identified six cases of vCJD. 2367 children had other diagnoses explain their deterioration. There were 259 other diseases in the diagnosed group. The paper provides unique epidemiological and clinical data about neurodegenerative diseases in children. The hope is that this information will facilitate earlier diagnosis in these complex cases.

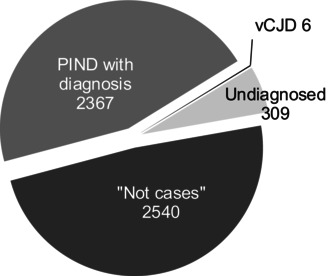

Plain language summary: https://onlinelibrary.wiley.com/doi/10.1111/dmcn.70067

**This original article is commented on by Elvidge on pages** 309–310 **of this issue.**

**Abbreviations**
BPSU: British Paediatric Surveillance Unit
NCJDSU: National Creutzfeldt–Jakob Disease Surveillance Unit
PIND: progressive intellectual and neurological deterioration
vCJD: variant Creutzfeldt–Jakob disease

**What this paper adds**

- Six children with variant Creutzfeldt–Jakob disease were notified, with no UK cases detected after 2003.
- There were 259 other neurodegenerative diseases affecting children with progressive intellectual and neurological deterioration.
- There was a relatively large proportion of Asian British children.
- Diagnosis was made before death in 99% of diagnosed children, while postmortems were increasingly rare.

The first report of bovine spongiform encephalopathy in British cattle was published in 1987^1^ and the National Creutzfeldt–Jakob Disease Surveillance Unit (NCJDSU) was established in Edinburgh in 1990. In 1996, Will and NCJDSU colleagues reported 10 cases of a new variant of Creutzfeldt–Jakob disease (CJD) (now referred to as variant CJD [vCJD]), with an earlier onset than sporadic CJD, a different clinical presentation, and a new neuropathological phenotype. Exposure to the bovine spongiform encephalopathy agent was suggested as the cause.^2^

There was concern that children in the UK might develop vCJD. There were two major difficulties in planning surveillance for vCJD among children: the clinical presentation might be different from that in adults and vCJD could be hidden among the many neurodegenerative diseases of childhood. Furthermore, the NCJDSU was led by adult neurologists who focused on the adult population. It was decided to carry out surveillance for children with progressive intellectual and neurological deterioration (PIND), defining a group of children that would include any with vCJD. The unique added value of the PIND study design was that it provided active prospective epidemiological surveillance of neurodegenerative diseases in children and young people in the UK. The methodology of the study has been described in previous publications.^3^, ^4^, ^5^, ^6^, ^7^, ^8^, ^9^, ^10^, ^11^, ^12^, ^13^, ^14^, ^15^

## METHOD

The study used the active national surveillance mechanism of the British Paediatric Surveillance Unit (BPSU), established in 1986,^16^ and was carried out in collaboration with the NCJDSU (later the National Creutzfeldt–Jakob Disease Research and Surveillance Unit).

When the study started, an orange surveillance card was posted by the BPSU to all UK consultant paediatricians. This was replaced by an online system, with up to a dozen rare disorders simultaneously under surveillance. Paediatricians reported eligible cases seen in the previous month. In 1997, more than 2000 cards were posted each month and over 90% were returned. Between 2016 and 2020 inclusive, the BPSU annual response rates varied between 88% and 94%.^17^

From May 1997, data were collected about notified children who met the case definition for PIND (Table 1) using a questionnaire, telephone interview with the notifying paediatrician, or site visit. Name and date of birth were needed to gather follow‐up information and investigation results from laboratories, some outside the UK. When the PIND study commenced in 1997, classification of ethnicity was that used by the Communicable Disease Surveillance Centre in the UK. The categories were: White, Black, Indian, Pakistani, Bangladeshi, Chinese, and Other; however, some were reported as ‘Asian’. For the analyses in this study, parents who were known to be first or second cousins were regarded as consanguineous.

**TABLE 1:** Case definition of progressive intellectual and neurological deterioration.

Any child under 16 years of age at onset of symptoms, who fulfils all of the following criteria: progressive deterioration for more than 3 months; loss of already attained intellectual or developmental skills; development of abnormal neurological signs.
**Excluding:** static intellectual loss; for example, after encephalitis, head injury, or near drowning.
**Including:** neurological disorders with specific diagnoses and meeting the case definition. Metabolic disorders leading to neurological deterioration; seizure disorders, but only if associated with progressive deterioration; known presymptomatic cases of diagnosed neurodegenerative conditions. Reporting was restricted to cases seen in the previous month but including those whose disorder began earlier (i.e. including ‘old cases’ of children at follow‐up if seen in that month).

A PIND study Expert Group, including specialists in paediatric neurology, metabolic diseases, and neurogenetics, plus a representative from the National Creutzfeldt–Jakob Disease Research and Surveillance Unit, met quarterly. It provided independent advice and made the final decision about the diagnoses, based on anonymized case details provided by the PIND study team. The notifying paediatricians were given detailed feedback, including suggestions for further investigations; the study follow‐up ceased when a diagnosis was made. Follow‐up of undiagnosed cases ceased when the patient died, when there were no plans for further investigations, or when the care was transferred to adult services.

The PIND team was not involved with patients or their families; it was agreed that cases with features suggestive of vCJD would be discussed with the local paediatrician, recommending referral to the National Creutzfeldt–Jakob Disease Research and Surveillance Unit.

Research ethics approval was obtained on 13th November 1996 from the Public Health Laboratory Service Ethics Committee (project no. 1996/20), and on 21st January 1997 from the Cambridge Local Research Ethics Committee (reference no. LREC97/10). On 14th February 2006, the Patient Information Advisory Group approved processing of patient identifiable information without consent; this approval was later obtained from the National Information Governance Board for Health and Social Care, the Public Health England Caldicott Advisory Panel, and the Public Benefit and Privacy Panel for Health and Social Care of Scotland.

## RESULTS

### Notifications

The study commenced in May 1997. After an initially large number of notifications of prevalent cases in the first 20 months of the study, from 1999 to 2018 inclusive the number settled to between 146 and 230 per year (relatively constant despite the COVID‐19 pandemic). From 2019 until active surveillance ceased at the end of 2023, it was 121 to 171 per year. After that, six cases were notified, the last in April 2024.

### Final classification of cases notified to the study

By April 2024, after 27 years, 5222 children had been notified and cases fell into the following groups. There were 2540 ‘not cases’, which included those not meeting the PIND case definition (48%), multiple notifications (26%), reporting errors (10%), and lack of information from the notifying paediatrician (16%) (this small subgroup could have included some cases with PIND). There were 309 children with PIND with no known diagnosis to explain their deterioration and 2373 children with an underlying diagnosis: six with vCJD (four definite and two probable) and 2367 others. The undiagnosed group and children with vCJD have been described in a recent article.^15^ The current article concentrates on the 2367 children with an underlying diagnosis other than vCJD.

The lifetime risk of having a diagnosed disease causing PIND was calculated by taking the 2373 diagnosed cases and dividing them by the number of total births in the diagnosis period, as suggested by Foss et al.^18^ The PIND study diagnosis period was May 1997 to April 2024. In the full years 1997 to 2023 inclusive, there were 19 598 293 live births in the UK^19^, ^20^, ^21^, ^22^, ^23^ This was taken as approximating to the PIND study diagnosis period; these births were used to calculate the lifetime risk of diagnosed PIND, which was 0.1 in 1000 live births. This is a conservative estimate because of the inevitable under‐ascertainment of cases and because undiagnosed children with PIND were not included but could have had neurodegenerative diseases. It should be noted that there are neurodegenerative diseases that do not meet the case definition for PIND.

### Demographics

Of the 2367 children who had diagnoses other than vCJD, 1265 were male and 1102 were female. Data about ethnicity was available for 2183 children. To compare the PIND study UK data with the 2021 census for England and Wales,^24^ the relevant ethnic groups were combined to produce the category ‘Asian’, that is, Asian British consisting of Pakistani (*n* = 449), Indian (*n* = 60), Asian (*n* = 56), Bangladeshi (*n* = 54), and Chinese (*n* = 6), for a combined total of 625. This was 28.6% of the 2183 children for whom data about ethnicity were available. The distribution of the other ethnic groups was: White 61.5% (1342 of 2183), Black 3.3% (72 of 2183), Mixed 3.1% (67 of 2183), and Other 3.5% (77 of 2183).

In the 2021 census^24^ for England and Wales, the percentage of the population in the Asian, Asian British, or Asian Welsh ethnic groups was 9.3%; White ethnic groups made up 81.7%; Black, Black British, Black Welsh, Caribbean or African made up 4.0%; mixed or multiple ethnic groups made up 2.9%; and other ethnic groups made up 2.1%.

The 2021 census data for England and Wales given in this article are not directly comparable to the PIND data collected from the whole UK between 1997 and 2023. However, the proportion of Asian British children in the PIND study at 28.6% is higher than the 9.3% of Asian British found in the general population of England and Wales. Autosomal recessive inherited diseases are a major cause of childhood PIND, so the relatively high rate of consanguinity in the PIND study Asian British group was not unexpected. Of the 548 children in this group for whom consanguinity information was available, 58% (317 of 548) had parents who were first or second cousins. Among Asian British children, the consanguinity rates for the individual ethnic groups were: Pakistani 67% (260 of 389); Asian 54% (26 of 48); Bangladeshi 48% (24 of 50); Indian 13% (7 of 55); and none in six Chinese children. In contrast, only 3% (27 of 1026) of the White group had parents who were first or second cousins.

### Investigations: neuropathology and molecular genetics

Brain biopsies were carried out during life in 14 children. In 13, they helped to make the diagnoses. These were: white matter disorders *n* = 4 (van der Knaap disease *n* = 2, Labrune syndrome *n* = 1, hypomyelination *n* = 1); Rasmussen encephalitis *n* = 3; mitochondrial diseases *n* = 2 (Alpers disease *n* = 1, Kearnes–Sayre syndrome *n* = 1); astrocytoma *n* = 1; herpes simplex encephalitis *n* = 1; infantile Batten disease *n* = 1; and multiple sclerosis *n* = 1.

In 1312 children, the results of molecular genetic investigations were available. In 864 cases (66% of 1312) these provided the diagnosis or confirmed it. Increasingly, cases were diagnosed using molecular genetic testing. The dates of testing were available for 573 of the 864 diagnostic results. In the first 5 years of the study (1997–2001 inclusive), molecular genetic testing contributed to the diagnosis in 32 children; in the last 5 years (2019–2023 inclusive), it contributed to the diagnosis in 206 children.

Once children had been diagnosed, systematic follow‐up by the PIND study ceased; however, by the end of the study in 2025, it was known that 1338 children had died (confirmed via the NHS England Digital Spine^25^). Of these, 39 had undergone postmortem investigations—in all but one, these were carried out before 2010. In 25 of 39 cases, the postmortem investigations were helpful in reaching the diagnosis, so the diagnosis was made before death in 2342 (2367–25) cases (99%).

Of the 25 diagnostic postmortem studies, one just had a liver biopsy (diagnosis: non‐ketotic hyperglycinaemia), and another just had a bowel biopsy (diagnosis: Degos disease). In the other 23, brain tissue was available for neuropathological study. The diagnoses were: mitochondrial diseases *n* = 8 (Alpers syndrome *n* = 5, Leigh syndrome *n* = 1, complex 1 deficiency *n* = 1, non‐specific *n* = 1); neurodegeneration with brain iron accumulation *n* = 4; white matter disorders *n* = 3 (vanishing white matter disease *n* = 1, van der Knaap disease *n* = 1, Alexander disease *n* = 1); pontocerebellar hypoplasia type 1 *n* = 2; astrocytoma *n* = 1; Aicardi–Goutières syndrome *n* = 1; dentato‐olivary dysplasia *n* = 1, GM1 gangliosidosis *n* = 1; Niemann–Pick type C *n* = 1; and Rasmussen encephalitis *n* = 1. Postmortem immunocytochemical staining of the brain for abnormal prion protein was carried out in just two cases.

### Distribution of diseases causing PIND

Excluding the 6 cases of vCJD, the diagnosed group consisted of 2367 children with 259 different diseases; different genotypes were counted as distinct diseases, as genetic studies revealed more specific diagnoses.

**FIGURE 1:**
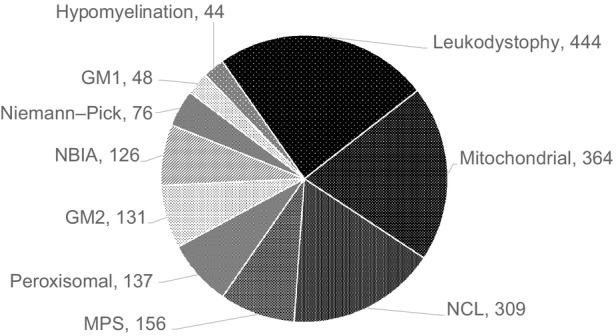
The 10 most common disease groups. The number of cases in each group is shown. In Table 2, 137 peroxisomal disorders are included in the 581 diagnosed leukodystrophies. Abbreviations: GM1, gangliosidosis type 1; GM2, gangliosidosis type 2; MPS: mucopolysaccharidosis; NBIA: neurodegeneration with brain iron accumulation; NCL: neuronal ceroid lipofuscinosis.

**FIGURE 2:**
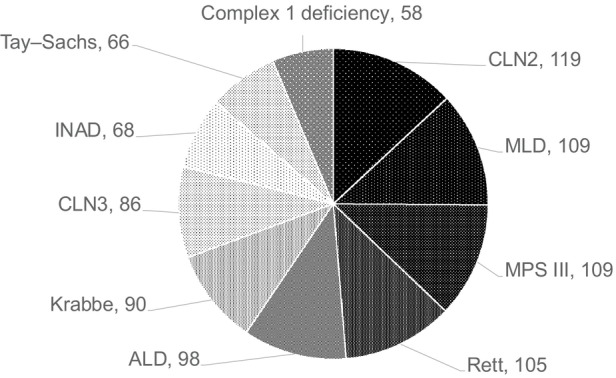
The 10 most common individual diseases. The number of cases in each group is shown. Abbreviations: ALD, adrenoleukodystrophy; CLN2, neuronal ceroid lipofuscinosis type 2 (late infantile); CLN3, neuronal ceroid lipofuscinosis type 3 (juvenile); INAD, infantile neuroaxonal dystrophy; MLD, metachromatic leukodystrophy; MPS III, mucopolysaccharidosis type III (Sanfilippo syndrome).

Table 2 summarizes the 259 diseases identified in the 2367 diagnosed children. Diagnostic mutations are shown where relevant. Of all the diseases, 157 of 259 (61%) were inborn errors of metabolism, which occurred in 1839 of 2367 (78%) of diagnosed children, showing that genetically determined (mainly autosomal recessive) diseases make a major contribution to PIND in children in the UK. Figure 1 shows the 10 largest diagnostic groups. The largest group consisted of 444 leukodystrophies (in Table 2, 137 peroxisomal disorders are included in the leukodystrophy group), the next of 364 mitochondrial diseases, followed by 309 neuronal ceroid lipofuscinoses (Batten disease). In comparison, Figure 2 shows the 10 most common individual diseases. Table 3 gathers together the 971 children (41% of the diagnosed group) who had 43 different lysosomal diseases.

**TABLE 2:** Summary of the diagnoses in 2367 children with PIND.

Individual disease or group of diseases	Individual diseases within the group	Number of cases
Arginase deficiency	–	7
Astrocytoma	–	3
Ataxia–telangiectasia	–	23
Biotinidase deficiency	–	6
Congenital disorders of glycosylation	Type 1a (*PMM2* mutation)	12
	Type 1 K	1
	*SLC35A*‐related	1
	*NGYLY1* mutation	1
Cobalamin C deficiency	–	9
Cockayne syndrome	Cockayne syndrome	23
	Connatal Cockayne syndrome type 2	1
3 hydroxyisobutyryl‐CoA hydrolase deficiency	–	3
Hydroxy glutaric aciduria	D‐2 hydroxy	3
	L‐2 hydroxy	6
DIDMOAD	–	4
DRPLA	–	3
Epileptic encephalopathy	Unverricht–Lundborg disease	2
	Lafora body disease	9
	Other	6
Ethylmalonic aciduria	–	3
Gaucher disease	–	5
Giant axonal neuropathy	–	6
Glutaric aciduria type 1	–	18
GM1 gangliosidoses	GM1 gangliosidosis (infantile, type I)	44
	GM1 gangliosidosis (juvenile, type II)	3
	GM1 gangliosidosis adult (type III)	1
GM2 gangliosidoses	GM2 gangliosidosis Tay–Sachs disease	66
	GM2 gangliosidosis juvenile Tay–Sachs disease	8
	GM2 gangliosidosis Sandhoff disease	50
	GM2 gangliosidosis juvenile Sandhoff disease	6
	GM2 gangliosidosis variant B	1
Huntington disease	–	29
Hypomyelination disorders	Non‐specific hypomyelination	19
	4H syndrome	5
	Leukodystrophy, *MAL* missense variant	2
	Hypomyelination with brainstem and spinal cord involvement	2
	Leukodystrophy (*DEGS1*)	1
	Leukodystrophy type 11	1
	Leukodystrophy type 6 (mutation in *TUBB4A*)	3
	Hypomyelination with cataracts (*FAM126A* deletion)	1
	Hypomyelinating leukodystrophy (*EIF2AK2*‐related)	1
	Unclassified leukoencephalopathy and hypomyelination	9
Inclusion‐cell disease	–	11
Lesch–Nyhan disease	–	5
Leukodystrophies	*Defects in genes encoding myelin protein*	
	Pelizaeus–Merzbacher disease	21
	*Disorders of amino/organic acid metabolism*	
	Canavan disease	26
	*Lysosomal storage disorders*	
	Metachromatic leukodystrophy	109
	Krabbe disease	90
	Multiple sulfatase deficiency	15
	Fucosidosis	4
	Free sialic acid storage disorder	1
	*Peroxisomal disorders*	
	Adrenoleukodystrophy	98
	Zellweger disease	17
	Peroxisomal biogenesis defect	10
	Infantile Refsum disease	5
	D‐bifunctional protein deficiency	7
	*Miscellaneous*	
	Aicardi–Goutières disease and variants	53
	Alexander disease	32
	Cerebrotendinous xanthomatosis	2
	Childhood‐onset progressive leukodystrophy (*ACER3* mutation)	1
	Hereditary spastic paraplegia variant with leukodystrophy	2
	Leukodystrophy and retinal dystrophy (*ACBD5* variant)	1
	Leukoencephalopathy with brainstem and spinal cord involvement	3
	Megalencephalic leukoencephalopathy with subcortical cysts (van der Knaap disease)	10
	Multiple sclerosis	3
	Vanishing white matter disease	29
	*Unclassified*	42
Leukoencephalopathies	Leukoencephalopathy (*IBA57*‐related) (cavitating)	1
	Leukoencephalopathy with brain calcifications and cysts (Labrune syndrome)	3
	Leukopathy with calcifications and cysts (CRMCC) (also known as Coats plus syndrome)	3
	Leukoencephalopathy H‐ABC syndrome (*TUBB4A* mutation)	1
	Sialidosis	1
Mannosidoses	–	6
Menkes disease	–	32
Methyltetrahydrofolate reductase deficiency	–	4
Mitochondrial diseases	Alpers syndrome (*POLG* mutations in 29)	37
	*DARS2* mutation	3
	Complex I deficiency	58
	Complex II deficiency	4
	Complex III deficiency	3
	Complex IV deficiency	20
	Kearnes–Sayre syndrome	5
	Leigh syndrome (25 specific diagnoses with 21 different mutations)	57
	MEGDEL syndrome	7
	MELAS syndrome	14
	MERFF syndrome	5
	Mitochondrial depletion syndrome	4
	Mitochondrial phenotype	39
	Multiple complex deficiency	9
	NARP syndrome	25
	Pyruvate dehydrogenase deficiency	27
	*RARS2* mutations	4
	*SURF1* mutations	18
	Miscellaneous (19 with diagnostic mutations)^a^	25
Molybdenum cofactor deficiency	–	14
Mucopolysaccharidoses	MPS type I Hurler syndrome	21
	MPS type II Hunter syndrome	25
	MPS type III Sanfilippo syndrome type A	87
	MPS type III Sanfilippo syndrome type B	18
	MPS type III Sanfilippo syndrome type C	2
	MPS type III Sanfilippo syndrome type D	2
	MPS unspecified	1
Mucolipidoses	Mucolipidosis type II	1
	Mucolipidosis type III (inclusion‐cell disease/pseudo‐Hurler polydystrophy)	1
	Mucolipidosis type IV	3
Neurodegeneration with brain iron accumulation	PLAN/INAD (*PLA2G6* mutation)	68
	PKAN (*PANK2* mutation)	43
	BPAN (*WDR45* mutation)	14
	Fatty acid hydroxylase‐associated neurodegeneration (*FA2H* mutation)	1
Neuronal ceroid lipofuscinoses	CLN1 (infantile Batten disease), 2 with GROD	45
	CLN2 (late infantile Batten disease)	119
	CLN3 (juvenile Batten disease) – 2 with GROD	86
	CLN5	10
	CLN6	16
	CLN7	14
	CLN8	8
	CLN14	1
	Variant	10
Niemann–Pick disease	Niemann–Pick type C (*NPC1*‐related)	66
	Niemann–Pick type A (*SMPD1*‐related)	6
	Niemann–Pick type A/B (*SMPD1*‐related)	4
Non‐ketotic hyperglycinaemia	–	19
Pontocerebellar hypoplasia	Type 1	2
	Type 2	3
	Type 3	1
Rasmussen encephalitis	–	11
Rett syndrome^b^	–	105
Subacute sclerosing panencephalitis	–	16
Succinic semialdehyde dehydrogenase deficiency	–	7
Sulphite oxidase deficiency	–	5
Thiamine disorder (*SLC19A3* mutation)	–	3
Wilson disease	–	8
*WWOX*‐related encephalopathy	–	3
Miscellaneous (72 different diseases)^a^	–	79

Abbreviations: BPAN, beta‐propeller protein‐associated neurodegeneration; CRMCC, cerebroretinal microangiopathy with calcifications and cysts; DIDMOAD, diabetes insipidus, diabetes mellitus, optic atrophy, and deafness (Wolfram syndrome); DRPLA, dentatorubral–pallidoluysian atrophy; GROD, granular osmiophilic deposit; MEGDEL, 3‐methylglutaconic aciduria with deafness, encephalopathy, and Leigh‐like syndrome; MELAS, mitochondrial encephalomyopathy with lactic acidosis and stroke‐like episodes; MERFF, myoclonic epilepsy with ragged‐red fibres; MPS, mucopolysaccharidosis; NARP, neuropathy, ataxia, and retinitis pigmentosa; PIND, progressive intellectual and neurological deterioration; PKAN, pantothenate kinase‐associated neurodegeneration (some reported as Hallervorden–Spatz disease, now a redundant term); PLAN/INAD, phospholipase A2‐associated neurodegeneration/infantile neuroaxonal dystrophy.

^a^ Miscellaneous groups consisted of diseases found in only one or two children and not included in the other diagnostic groups in the table.

^b^ Not all cases of Rett syndrome fit the definition of PIND. Some of the cases were diagnosed according to phenotype; in others, diagnostic mutations were found.

**TABLE 3:** Lysosomal storage disorders (971 cases, 43 diseases)^a^.

Group	Individual diseases	Number of cases
Cystinosis	–	1
Fucosidosis	–	4
Galactosialidosis	–	1
Gaucher disease	–	6
GM1 gangliosidoses	GM1 gangliosidosis (infantile, type I)	44
	GM1 gangliosidosis (juvenile, type II)	3
	GM1 gangliosidosis adult (type III)	1
GM2 gangliosidoses	GM2 gangliosidosis Tay–Sachs disease	66
	GM2 gangliosidosis juvenile Tay–Sachs disease	8
	GM2 gangliosidosis Sandhoff disease	50
	GM2 gangliosidosis juvenile Sandhoff disease	6
	GM2 gangliosidosis variant B	1
Inclusion‐cell disease	–	11
Krabbe disease	–	90
Leukodystrophies	Metachromatic leukodystrophy	109
	Salla disease (free sialic acid storage disease)	1
Leukoencephalopathy	Sialidosis 1	1
Mannosidoses	–	6
Mucopolysaccharidoses	MPS type I Hurler syndrome	21
	MPS type II Hunter syndrome	25
	MPS type III Sanfilippo syndrome type A	87
	MPS type III Sanfilippo syndrome type B	18
	MPS type III Sanfilippo syndrome type C	2
	MPS type III Sanfilippo syndrome type D	2
	MPS unspecified	1
Mucolipidoses	Mucolipidosis type II	1
	Mucolipidosis type III (inclusion‐cell disease/pseudo‐Hurler polydystrophy)	1
	Mucolipidosis type IV	3
Multiple sulfatase deficiency	–	15
Neuronal ceroid lipofuscinosis	CLN1 (infantile Batten disease), 2 with GROD	45
	CLN2 (late infantile Batten disease)	119
	CLN3 (juvenile Batten disease), 2 with GROD	86
	CLN5	10
	CLN6	16
	CLN7	14
	CLN8	8
	CLN14	1
	Variant	10
Niemann–Pick disease	Niemann–Pick disease type C (*NPC1*‐related)	66
	Niemann–Pick disease type A (*SMPD1*‐related)	6
	Niemann–Pick disease type A/B (*SMPD1*‐related)	4
Schindler disease	–	1

Abbreviations: GROD, granular osmiophilic deposit; MPS, mucopolysaccharidosis.

^a^ These disorders are included in Table 2.

Children with PIND first presented to the notifying paediatricians with relatively non‐specific clinical features. In 2024, after 26 years of surveillance, 2335 children were in the group with a diagnosis. Their presenting symptoms and signs are as follows: slow development 814 (35%); developmental regression or cognitive decline 349 (15%); seizures 317 (14%); motor dysfunction (e.g. ataxia, gait problems) 311 (13%); visual abnormality or deterioration 115 (5%); positive family history 114 (5%); dysmorphic features 69 (3%); neonatal problems 64 (3%); acute encephalopathy 50 (2%); and others 132 (6%).

## DISCUSSION

This study carried out surveillance for 27 years, starting in May 1997. It was designed to capture cases of vCJD by reviewing all notified children in the UK with PIND and thus provided reassurance that just six cases of vCJD occurred in children; the last two died in 2003.^15^ Unique data were acquired about the 2367 children with PIND who had 259 different underlying diagnoses (other than vCJD) to explain their deterioration. A previous paper^14^ reported on the proportion of cases that presented in four different age groups (<1 year 40%, 1–4 years inclusive 41%, 5–9 years inclusive 13%, 10–15 years inclusive 6%) and gave details of the most common diseases found in those age groups in White and Pakistani children (see below).

### Changes in the investigation of neurodegenerative diseases

The study has charted the evolution of investigations since 1997. Brain biopsies were performed in 14 diagnosed children and they helped to make a diagnosis in 13. Four of the six children with vCJD had postmortem neuropathology.^15^ Only 39 of the other 1338 diagnosed children who died were known to have undergone postmortem investigations. These contributed to the diagnosis in 25 cases; however, all but one were carried out before 2010 and in 99% of the 2367 cases the diagnosis was made before death.

Increasingly, cases with PIND were diagnosed according to genotype. For instance, there were 57 cases of Leigh disease, 32 diagnosed on the basis of phenotype; in the other 25, there was a specific diagnosis (21 different mutations were found). Table 2 includes the cases with PIND diagnosed according to phenotype and also those where a diagnostic mutation was found.

In 1312 children, the results of their molecular genetic investigations were available; 66% of these provided the diagnosis or confirmed it, an increasing proportion with time. Sometimes diagnoses were made using gene panel testing or because cases had been entered into the 100000 Genome Project^26^ or the Deciphering Developmental Disorders Study.^27^ However, in most, the diagnosis was reached in a more targeted way by using the phenotype and the results of key investigations; for example, brain magnetic resonance imaging and enzyme studies, followed by genetic testing where relevant.

### Ethnicity and consanguinity

In 2004, the study reported that in some UK health districts there were unexpectedly high numbers of cases with PIND, with a heterogeneous mixture of underlying diagnoses. In the three districts with the largest numbers of resident cases, most not only came from families of Pakistani or Bangladeshi ancestry but also had high reported rates of consanguinity.^4^ In 2021, the study reported on the differential diagnosis of PIND in children in the UK. The two largest ethnic groups were White and Pakistani (58.2% and 17% of diagnosed cases respectively). The most common diseases in these two ethnic groups were shown for the four age groups described above. The distribution of diseases varied with age but was quite similar in some White and Pakistani children.^14^ The current study provides a conservative estimate of consanguinity rates because the analysis included only children whose parents were reported to be first or second cousins. The findings support the conclusions reached by Corry,^28^ who studied consanguinity and prevalence patterns of inherited disease in the Pakistani community in the UK. His analysis suggested that much of the increased mortality and morbidity in children of Pakistani ancestry in the UK was due to autosomal recessive conditions and that this finding was associated with the custom of consanguineous marriage. Two later studies of consanguineous Pakistani families using molecular genetic techniques identified many new candidate genes in children with autosomal recessive neurodevelopmental disorders in the families.^29^, ^30^

### Other studies of progressive intellectual and neurological deterioration in children

The case definition for this study was designed to capture any cases of vCJD and delineated a subgroup of all children with neurodegenerative diseases. Therefore, it is not surprising that there are relatively few publications that report PIND in children. The Canadian Paediatric Surveillance Program studied Canadian children and young people aged 18 years or younger between July 1999 and 2001.^31^ Of the notified cases, 60 had a progressive neurological syndrome associated with intellectual deterioration. Their findings were described as being similar to those of the PIND study, with the important difference that no vCJD cases were identified.

Warmerdan et al.^32^ performed a literature search on inborn errors of metabolism presenting with PIND in individuals aged 0 to 18 years. They identified 85 inborn errors of metabolism, concluding that inborn errors of metabolism constitute the largest group of PIND conditions, referring to four PIND study papers,^3^, ^4^, ^8^, ^13^ to support this statement.

Elvidge et al.^33^ performed a literature review of conditions that met their case definition for childhood dementia and their findings overlapped with those of the PIND study. One hundred and seventy genetic childhood dementia disorders were identified. Untreatable childhood dementia was estimated to have an incidence of 34.5 in 100 000. The authors compared this to the incidence of UK cases with PIND (10 in 100 000 live births^14^) and the estimated incidence rates of progressive encephalopathy in Norway^34^ and Sweden^35^ of 60 and 58 in 100 000 live births respectively. They noted that the Australian Childhood Dementia Study^36^ reported that the cumulative 2‐year prevalence of dementia for children aged under 15 years was 5.6 in 100 000. The disparity between these estimates reflects the differences in case definitions and methodologies between these studies.

### Study strengths

Surveillance was carried using the BPSU, which had been very successful in studying rare diseases in UK children and was valued by paediatricians, with good response rates to studies.^16^

The National Institute for Health and Care Research (NIHR) PIND study grant funded two full‐time‐equivalent public health researchers, who were able to discuss cases with notifying paediatricians and if necessary carry out a site visit to extract clinical data from the hospital notes. Approval was obtained from the relevant regulatory bodies to obtain patient identifying information (e.g. name, date of birth, home postcode) without prior parental or carer consent. Previous public health surveillance experience showed that the need to obtain written consent before sharing data made studies impractical.^37^

Cases meeting the criteria for PIND were reviewed by the members of the PIND study Expert Group, who provided independent evaluation of all the diagnoses made in notified children and gave feedback to the paediatricians who notified them.

### Study limitations

The study depended on the voluntary involvement of the paediatricians who notified cases; in all BPSU studies, there has been under‐ascertainment of eligible cases because of the time involved in completing questionnaires and providing follow‐up information. However, in 2004, the PIND study reported on the different numbers of cases (according to residence) in the 126 Public Health Laboratory Service UK health districts. Only six health districts had not reported any cases with PIND, demonstrating widespread support for the study.^4^

There were some grey areas in the interpretation of the case definition of PIND. Examples are: paediatricians did not always realize that they should notify cases if an underlying diagnosis had already been made; seizure disorders were not usually included but some genetically determined seizure syndromes met the criteria for PIND; children with Rett syndrome met the criteria for PIND when they deteriorated early in life but not later when they were more stable.

Notified patients were not followed up by the study after a diagnosis was made. In undiagnosed cases, follow‐up ceased when no further investigations were planned, when the child died, or when care had been transferred to adult services. Occasionally, there was subsequent information that a diagnosis had been made. However, the study did not provide systematic long‐term follow‐up.

## CONCLUSIONS

The PIND study carried out active prospective surveillance of a carefully defined group of neurodegenerative diseases for 27 years. It provided reassurance that cases of vCJD were not being missed in children in the UK,^15^ and collected unique epidemiological and clinical data about the geographical and ethnic distribution of this complex group of diseases.^4^, ^14^ It has provided a wealth of information about the mode of presentation, clinical features, and modes of diagnoses in many different rare neurodegenerative diseases.^7^, ^10^, ^11^, ^12^ It demonstrated the reappearance of subacute sclerosing panencephalitis in association with increasing measles infections secondary to falling measles vaccination rates.^38^ Throughout the study, the PIND study Expert Group gave specialist advice, which was valued by the paediatricians who notified cases. This was a productive interaction that provides a model for the future management of this complex group of disorders. Increasingly, these diseases are treatable and it is hoped that the clinical information gathered by the PIND study will facilitate earlier diagnosis in these challenging cases.

## Data Availability

The data that support the findings of this study are available on request from the corresponding author. The data are not publicly available due to privacy or ethical restrictions.
